# Validation of the Arabic version of the composite autonomic symptom score 31 questionnaire in diabetic autonomic neuropathy

**DOI:** 10.1007/s10072-026-08990-w

**Published:** 2026-03-27

**Authors:** Ahmed S. Alkotami, Asmaa Aboelfath, Aya E. Zhran, Amro M. Stino, Ahmed M. Eldokla

**Affiliations:** 1 Department of Neurology, Tanta University, Tanta, Gharbia Egypt; 2 Department of Neurology, Mansoura University, Mansoura, Dakahlia, Egypt; 3 Department of Neurology, University of Michigan, Ann Arbor, MI USA; 4 Department of Neurology, Division of Neuromuscular Medicine, The University of Michigan, F2647 UH South, 1500 E. Medical Center Drive, Ann Arbor, MI 48109- 5223 USA; 5 NY Neuromuscular Healthcare, Syracuse, NY 13205 USA

**Keywords:** COMPASS-31, Validation, Diabetic autonomic neuropathy, Autonomic dysfunction, Quality of life, Arabic translation

## Abstract

**Background:**

Diabetic autonomic neuropathy (DAN) is a significant complication of diabetes. The Composite Autonomic Symptom Score-31 (COMPASS-31) is a widely used and validated instrument to assess autonomic dysfunction, yet no validation of its use exists in the Arabic language. In this study, we aimed to develop and validate an Arabic language version of the COMPASS-31 (A-COMPASS-31) in a cohort of Egyptian patients with DAN.

**Methods:**

We enrolled 20 diabetic subjects with DAN and 20 age- and sex-matched diabetic subjects without DAN. Following International Society for Pharmacoeconomics and Outcomes Research (ISPOR)-guided adaptation, the A-COMPASS-31 was developed and subsequently administered twice (four weeks apart) for test-retest reliability. Convergent validity was evaluated against the Ewing battery of autonomic tests, sympathetic skin response (SSR), the neuropathic pain scale (NPS), somatic neuropathy (The Utah Early Neuropathy Scale (UENS)), and quality of life (EuroQol-5 Dimensions-5 Levels (EQ-5D-5 L)) measures. Receiver operating characteristic (ROC) analysis determined diagnostic accuracy.

**Results:**

A-COMPASS-31 demonstrated excellent reliability (Cronbach’s α = 0.841; ICC = 0.992). It significantly correlated with the UENS (r_s_=0.517, *p* = 0.001), NPS (r_s_=0.495, *p* = 0.001), and EQ-5D-5L (r_s_=-0.539, *p* = 0.001). ROC analysis yielded fair diagnostic accuracy (AUC = 0.742, 95% CI 0.580–0.867, *p* = 0.003) with an optimal cut-off of 28.43 (sensitivity 80%, specificity 65%).

**Conclusion:**

The A-COMPASS-31 is a reliable and valid instrument for assessing autonomic symptoms in Arabic-speaking diabetic patients. Its strong correlation with neuropathy severity scales and quality of life measures supports its utility as a first-line clinical screening tool. Due to its modest specificity, initial screening should be followed by objective autonomic testing for definitive diagnosis.

**Supplementary Information:**

The online version contains supplementary material available at 10.1007/s10072-026-08990-w.

## Introduction

Diabetic autonomic neuropathy (DAN) is a disabling complication of diabetes that presents subclinically in up to 20% of patients [1]. Cardiovascular autonomic neuropathy (CAN) is the most extensively researched and serious presentation of DAN, and associates with increased risk of cardiovascular injury, cardiac wall remodeling, and atypical ischemic heart disease [2], leading to increased morbidity and mortality. Prevalence figures for CAN among diabetics vary widely, ranging from 17% to 73% [3].

The Ewing battery of tests of autonomic function have long been considered a gold standard for CAN diagnosis [4, 5], and offer a practical alternative to the full autonomic reflex screen in resource-constrained environments. In addition, the sympathetic skin response (SSR) is an electrophysiological test that measures skin conductance changes caused by the activation of the sympathetic nervous system, and can be used to assess small C unmyelinated nerve fibers [6, 7]. While formal autonomic testing via the Ewing battery or sympathetic skin response can be employed, testing can be time-consuming and requires specialized equipment and technical expertise, limiting widespread adoption [8, 9].

To that end, the use of self-administered questionnaires evaluating autonomic symptoms provides an easy and valuable initial screening option. The Composite Autonomic Symptom Score-31 (COMPASS-31) is a widely used and validated tool that evaluates autonomic dysfunction across six domains [10]. The COMPASS-31 carries great value as a rapid first-line screening tool for DAN both in diabetic and primary care clinics [11–13]. It has been translated and validated in multiple languages, including Croatian and Serbian [14], Danish [15], German [16], Italian [17], Korean [18], Norwegian [12], Thai [19] and Turkish [20]. To date, however, it has not been translated or validated in Arabic. This carries practical importance, as Egypt, for one, ranks amongst the top ten countries in the world for diabetes [21], with the Middle East North Africa (MENA) region expected to have > 100 m diabetics by 2045 [22], many of whom are not being sufficiently screened for DAN.

Given that linguistic and cultural factors significantly influence symptom reporting, the development of a culturally adapted Arabic version is essential for accurate clinical assessment in this demographic. Our study aimed to address this unmet need by developing and validating an Arabic language version of COMPASS-31. We also sought to correlate individual COMPASS-31 domains and total score with the Ewing battery of individual tests, the sympathetic skin response (SSR), the neuropathic pain severity (NPS), the Utah Early Neuropathy Scale (UENS) [23], and the EuroQol-5 Dimensions-5 Levels (EQ-5D-5 L) index value, as adjusted for the Egyptian population [24].

## Subject and methods

### Translation process of the COMPASS-31 from English into Arabic

The translation process adhered strictly to the guidelines established by the International Society for Pharmacoeconomics and Outcomes Research (ISPOR) for the cross-cultural adaptation of patient-reported outcome measures. After obtaining permission from the original COMPASS-31 authors, two of our authors (AE and AA), both of whom are neurologists with subspecialty expertise in autonomic disorders, as well as native Arabic speakers with English proficiency, independently translated the original English version of the COMPASS 31 questionnaire [10] into Arabic. AE had previous translated an Arabic version of the questionnaire [25], and both AE and AA produced a unified version. This version was then translated back into English by an independent, bilingual medical professional blinded to the original questionnaire, to ensure its alignment with the original English version. This process culminated in the generation of one final Arabic version of the questionnaire. The study team reviewed the final version in relation to the original, identifying any semantic inconsistencies or items requiring refinement.

### Study subjects

Study participants were recruited from the outpatient neuromuscular clinic at Tanta University Hospitals and Center of Neurology and Psychiatry in Tanta, Egypt between March 2025 and June 2025. This study was approved by The Research Ethics Committee at Tanta University. All participants provided informed consent, and participation was completely voluntary.

We enrolled 20 patients with a confirmed diagnosis of diabetes (26) (previously diagnosed with A1c ≥ 6.5% or fasting glucose ≥ 126 mg/dL ) and DAN. DAN was defined based on the presence of either two abnormal heart rate tests of Ewing battery or absent Sympathetic Skin Response (SSR). In addition, we enrolled 20 age and sex matched diabetic patients, similarly defined, but who did not have DAN. These patients did not report neuropathic symptoms (e.g., numbness or tingling), and clinical examination revealed no evidence of neuropathy. In addition, these subjects had a normal Ewing battery of tests and a normal SSR response. Subjects were re-interviewed for the Arabic version of the COMPASS-31 (A-COMPASS-31) twice for the assessment of test-retest reliability, with an inter-test interval of 4 weeks to minimize recall bias. To avoid any potential influence on responses to the questionnaire, subjects were not informed of the results of the Ewing battery or SSR until completion of the questionnaire both times.

### COMPASS-31 scoring system

The COMPASS-31 is a validated, 31-item self-assessment questionnaire that assesses autonomic symptoms in six domains: orthostatic intolerance, vasomotor, secretomotor, gastrointestinal, bladder, and pupillomotor functions. Each subdomain has a maximum weighted score for dysfunction: 40 for orthostatic intolerance, 5 for vasomotor, 15 for secretomotor, 25 for gastrointestinal, 10 for bladder, and 5 for pupillomotor. A raw domain score was calculated by adding scores from each domain. Each domain total score was weighted (i.e., total domain score was multiplied by a weighing factor) and then formatted to yield a total score ranging from 0 to 100 [10].

### Objective definition of diabetic autonomic neuropathy

To fulfill objective criteria for DAN, subjects were required to demonstrate objective evidence of autonomic dysfunction in either the cardiovascular or sudomotor domains. CAN was defined by the presence of at least two abnormal heart rate tests from the Ewing battery. Alternatively, sudomotor autonomic dysfunction was defined by an abnormal (absent) SSR.

### Ewing battery of tests

The Ewing battery includes heart rate variation during deep breathing, the Valsalva maneuver, heart rate response to standing, and blood pressure response to standing, as well as sustained handgrip. Not all components of the Ewing battery, however, are required to define CAN. To define CAN for our study purposes, at least two of three heart rate tests performed (as part of the Ewing battery) were required to be abnormal [4, 5, 26].

#### Heart rate tests

Heart rate testing was conducted via a DEYMED TruTrace^®^ EMG device (DEYMED Diagnostic, Hronov, Czech Republic) using the R-R interval analysis. While the patient rested supine, continues ECG was recorded by placing the active electrode on the left wrist, the reference electrode on the right wrist (at sites of median nerve stimulation), with the ground electrode on the dorsum of the left hand. Filters were adjusted such that low frequency was set at 3–5 Hz and high frequency 30–40 Hz, with notch used if necessary. Gain was adjusted from 300 to 700 µV per division as necessary, and the sweep duration was 500 ms per division. Recording duration was 60 s (equal to 12 sweeps).

1. The 30:15 ratio

The 30:15 ratio is the R-R interval at the 30th beat (the slowest heart rate) divided by interval at the 15th beat (fastest heart rate), measured during the first 30–45 s of active standing. The 30:15 ratio was considered normal if ≥ 1.04, borderline if 1.01–1.03, and abnormal if ≤ 1.00 [4, 27, 28].

2. Valsalva maneuver

The Valsalva maneuver was performed by asking the patient to blow into a mouthpiece connected to a simple aneroid sphygmomanometer gauge, at an average pressure of 40 mmHg for 15 s. Expiration was initiated and terminated abruptly. The Valsalva ratio (VR) was calculated by dividing the longest R-R interval shortly after the maneuver to the shortest R-R interval during the maneuver. Normally, the ratio is ≥ 1.21, with a borderline range between 1.11 and 1.20, and abnormal values ≤ 1.10 [4, 29].

3. Heart rate response to deep breathing

Heart rate response to deep breathing was performed by asking the patient to breathe deeply and evenly at a rate of six breaths per minute, with each inhalation and exhalation phase lasting 5 s, without any pause or gasp. The maximum duration of the maneuver was 1 min. Expiratory to inspiratory (E: I) ratio was calculated by measuring the ratio of the longest R-R interval during expiration (RRmax) to the shortest R-R interval during inspiration (RRmin). Normal E: I ratio is ≥ 1.21 with a borderline range between 1.11 and 1.20, and abnormal values ≤ 1.10. Breathing ratio was calculated by the device as (RRmax - RRmin)/RRaverage [4, 8].

#### Blood pressure tests

Blood pressure measures were assessed using a calibrated Beurer BM-28 blood pressure monitoring device, which was previously validated according to the International Protocol of the European Society of Hypertension (ESH-IP) revision 2010 [30].

1. Diastolic blood pressure response to sustained hand grip

Blood pressure was measured every minute over 3 min in the non-tested arm, while the patient compressed a hand dynamometer (Handexer™ Grip Strength Tester, California, USA) to 30% of the maximum force of fist closure. The difference between diastolic blood pressure just before the release of the handgrip and before starting was taken as the measure of the response. The normal response was defined as an increase in diastolic BP ≥ 16 mmHg, with a borderline value of 11–15 mmHg increase, and an abnormal value defined as an increase ≤ 10 mmHg [31].

2. Systolic blood pressure to active standing

The patient lied supine for at least 5 min during which blood pressure was monitored once or twice. Then, the patient was asked to actively stand upright and blood pressure was measured within 1–3 min of standing. The normal response was defined as a drop in systolic blood pressure ≤ 10 mmHg, with a borderline drop defined as 11 to 29 mmHg, and an abnormal drop defined as ≥ 30 mmHg [31].

### Sympathetic skin response (SSR)

The SSR test was performed to assess C nerve fiber function by evaluating sudomotor function. We performed bilateral simultaneous two-channel hand-to-hand stimulation by stimulating the median nerve on one side and recording from the other side. Foot-to-foot stimulation was similarly performed by stimulating the tibial nerve on one side and recording from the other. In hand stimulation, the recording electrodes were applied to the palms of either hand, the reference electrodes were applied to the dorsa of either hand, and the ground electrode was applied to the forearm of the stimulated side. In foot stimulation, the recording electrodes were applied to the soles of either foot, the reference electrodes were applied to the dorsa of either foot, and the ground electrode was applied to the foreleg of the stimulated side. The sensitivity was adjusted as needed from 200 to 3000 µV/div, and the filters were set at 0.2 Hz and 15 Hz for the low- and high-frequency filters, respectively. Stimulation was applied at randomized intervals of variable intensity (8–60 millivolts and duration up to 500 milliseconds) to prevent any habituation effects. An absent SSR response in at least one lower limb was considered abnormal [6, 32].

## Neuropathy and quality of life scales

### Neuropathic pain scale

The NPS measures neuropathic pain intensity, scored from 0 to 10, with 0 being “no pain” and 10 being “the worst pain imaginable” [33].

### Utah early neuropathy scale (UENS)

The UENS is a validated instrument for evaluating neuropathy, especially small nerve fiber injury, with total scores ranging from 0 to 42. It primarily evaluates length-dependent affection of pinprick sensation at six sites on each leg and foot, assigning a score of 1 for diminished sensation and 0 for absent sensation. The scale also evaluates allodynia, the integrity of large sensory nerve fibers (great toe vibration and position testing), and motor function (assessed through great toe dorsiflexion) [23].

### The 5-level european quality of life-5 dimensions scale (EQ-5D-5 L)-Arabic version

The EQ-5D-5 L Arabic version was used to assess quality of life. It is a self-reported questionnaire that assesses quality of life in five dimensions: mobility, self-care, usual activities, pain/discomfort, and anxiety/depression. Each dimension has five response levels ranging from 1 to 5, extending from no problems (1) to inability to function / extreme problems (5). The final response is then converted to a single utility index developed through a standardized valuation study protocol (EQ-VT v2.0) that was developed by the EuroQol group [24], and using country-specific cutoffs for Egypt.

### Statistical analysis

For numerical values, the Shapiro–Wilk test was performed to assess normal distribution. For parametric variables, mean and standard deviations (SD) were calculated. For non-parametric variables, median and interquartile range percentiles (IQR) were calculated. The Mann-Whitney test (U) was used to compare two numerical groups. For categorical variables, the number and percentage were calculated and differences between subcategories were tested by chi-square test.

Internal consistency of COMPASS-31 scores was calculated using the Cronbach’s α and McDonald’s ω, and test-retest reliability was assessed by the intraclass correlation coefficient (ICC) model (3,k) for test subdomains and total score, with the Bland-Altman plot used for the total scores. Convergent validity was assessed by correlation between the COMPASS-31 scores and the different autonomic function tests, somatosensory scales, and quality of life EQ-5D-5 L index score. Correlation between numerical values was performed using Spearman’s correlation (r_s_), while associations between categorical and continuous data used point biserial correlation (r_bp_). To account for multiple testing and control the risk of Type I error, the Benjamini-Hochberg procedure was applied to control the False Discovery Rate (FDR) for our primary convergent validity analyses (correlations involving the total A-COMPASS-31 score). Because not all symptom sub-domains are physiologically expected to correlate with specific cardiovascular or sudomotor reflexes, correlations involving individual COMPASS-31 sub-domains were treated as exploratory to avoid artificially inflating the multiple-testing penalty. A receiver operating characteristic (ROC) curve was generated to assess the sensitivity, specificity, and cut-off values for total COMPASS-31 scores in diagnosing DAN.

In planning the study, sensitivity power analysis was conducted to evaluate the adequacy of the study sample. For correlation analyses, the minimum detectable Spearman correlation at 80% power with *n* = 40 and α = 0.05 (two-tailed) was calculated using Fisher’s z-transformation. For the ROC analysis, power was computed using the Obuchowski method. For the between-group comparison for the total A-COMPASS-31 score, power was calculated based on Cohen’s d for independent two-sample t-tests. To quantify the precision of the estimates, 95% confidence intervals for Spearman correlations were computed using Fisher’s z-transformation, and for Cohen’s d using the Hedges and Olkin approximation (Supplement Table S1).

For all statistical tests done, the threshold of significance was fixed at a p value ≤ 0.05. All data were organized, tabulated, and statistically analyzed using jamovi version 2.6, with R version 4.4.2.

## Results

The study population consisted of a total of 40 participants, divided equally into 20 diabetic subjects with confirmed DAN and 20 diabetic subjects without DAN. The groups were well-matched for age (50.5 ± 7.19 vs. 49.25 ± 6.58 years; *p* = 0.570), sex, height, and body mass index (BMI) (Table 1). However, patients with confirmed DAN exhibited a significantly higher metabolic burden, characterized by increased waist circumference (*p* = 0.046), higher resting systolic blood pressure (*p* = 0.032), and a significantly higher prevalence of metabolic syndrome (68% vs. 32%; *p* = 0.003).

**Table 1:** Demographic and clinical characteristics of the subjects studied

Variable	Diabetic subjects with DAN (*n* = 20)	Diabetic subjects without DAN (*n* = 20)	*p*
Age (years)	50.50 ± 7.19	49.25 ± 6.58	0.570
Sex (Female)	14 (70%)	16 (80%)	0.465
Weight (kg)	90.47 ± 16.49	85.37 ± 15.96	0.326
Height (m)	1.63 ± 0.10	1.61 ± 0.07	0.506
BMI (kg/m^2^)	34.07 ± 6.22	32.77 ± 5.58	0.489
Waist Circ. (cm)	109.4 ± 14.06	99.90 ± 15.03	0.046*
Systolic BP (mmHg)	131.9 ± 11.80	122.15 ± 15.60	0.032*
Diastolic BP (mmHg)	80.10 ± 9.90	74.60 ± 12.31	0.128
HbA1C	8.20 ± 1.69	6.12 ± 1.23	< 0.001*
Metabolic syndrome	17 (68%)	8 (32%)	0.003*

*DAN* Diabetic autonomic neuropathy, *BMI* Body mass index, *Circ* circumference, *BP *Blood pressure

* Significant. Data are presented as mean ± SD, or number (percentage)

**Fig. 1:**
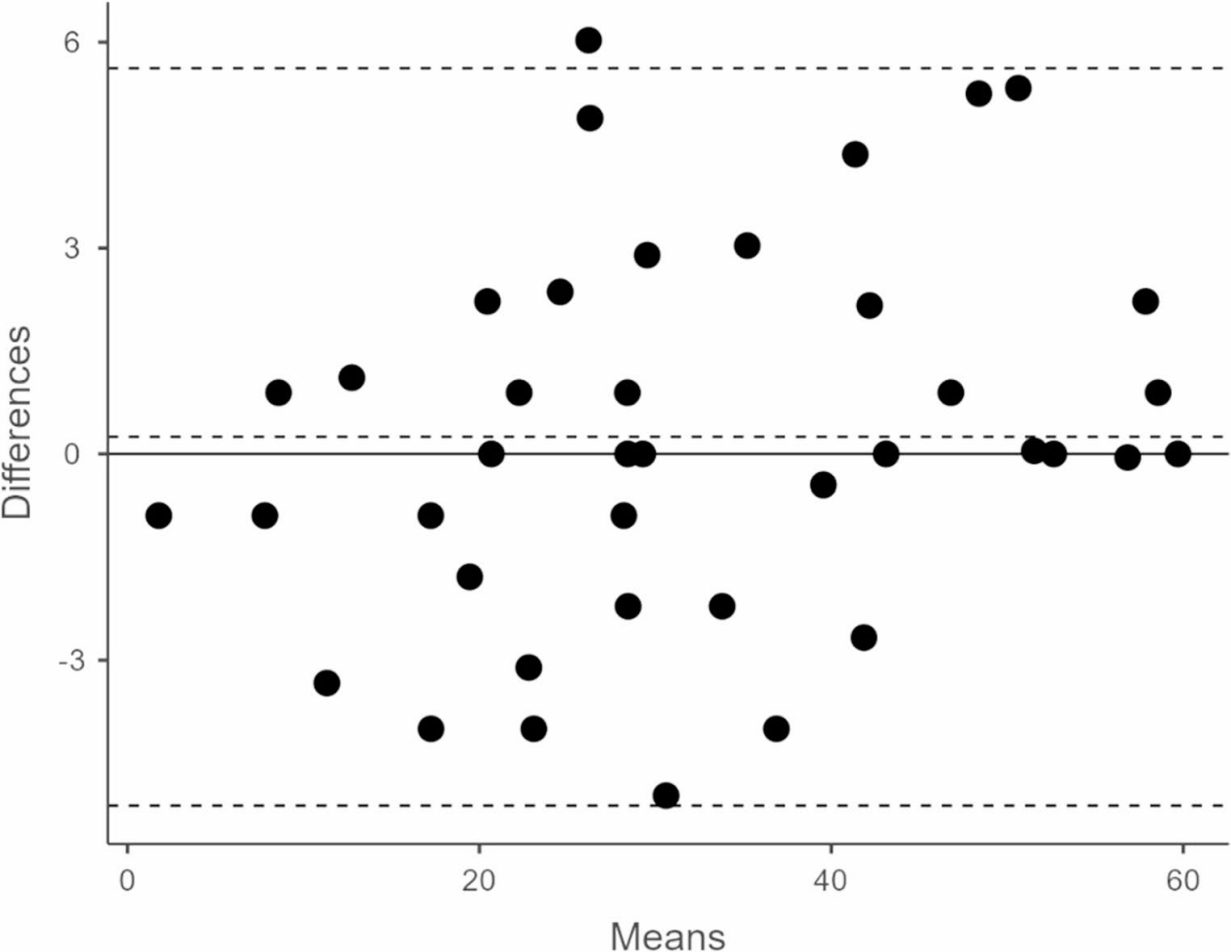
Bland-Altman plot for total COMPASS-31 test-retest reliability

The Arabic COMPASS-31 demonstrated strong internal consistency with a total Cronbach’s α of 0.841 and McDonald’s ω of 0.8443. Domain-specific analysis showed high consistency (α > 0.70) for most subscales (Table 2). The gastrointestinal domain initially showed lower consistency (α = 0.615); however, excluding the diarrhea-related items (questions 16–19) improved the domain’s α to 0.7114. Test-retest reliability was excellent, with an ICC of 0.992 for the total score5. The Bland-Altman plot confirmed high stability, showing a mean difference close to zero with narrow limits of agreement (Fig. 1).

**Table 2:** Internal consistency and test-retest reliability of Arabic COMPASS-31 scores

COMPASS-31 (weighting factor)	Raw score	Weighted score	Cronbach’s α	McDonald’s ω	ICC
Total score	22.5 (13.75-29)	29.04 (21.21–43.33)	0.841	0.844	0.992
DAN +ve	27.5 (21-30.5)	39.92 (28.66–51.15)			
DAN -ve	14.5 (10.75–23.5)	24.24 (16.4-31.41)			
p	0.004*				
Orthostatic intolerance (4)	4 (2–6)	16 (8–24)	0.854	0.889	0.988
DAN +ve	5 (3.75–6.25)	20 (15–25)			
DAN -ve	3 (2-5.25)	12 (8–21)			
P	0.102				
Vasomotor (0.83333333)	0 (0–3)	0 (0-2.5)	0.908	0.913	0.995
DAN +ve	2 (0–3)	1.67 (0-2.5)			
DAN -ve	0 (0–3)	0 (0-2.5)			
p	0.146				
Secretomotor (2.1428571)	2 (0–3)	4.29 (0-6.43)	0.526	0.610	0.982
DAN +ve	3 (2–3)	6.43 (4.29–6.43)			
DAN -ve	0 (0–3)	0 (0-6.43)			
p	0.008				
Gastrointestinal (0.8928571)	8 (4.75-10)	7.14 (4.24–8.93)	0.615**	0.686**	0.980
DAN +ve	8 (6.75-11)	7.14 (6.03–9.82)			
DAN -ve	7.5 (2.75–9.25)	6.7 (2.46–8.26)			
p	0.237				
Bladder (1.1111111)	1.5 (0–3)	1.67 (0-3.33)	0.757	0.826	0.889
DAN +ve	3 (0–3)	3.33 (0-3.33)			
DAN -ve	1 (0–2)	1.11 (0-2.22)			
p	0.05				
Pupillomotor (0.3333333)	5 (3-8.25)	1.67 (1-2.75)	0,734	0.746	0.999
DAN +ve	7 (5.25-9)	2.33 (1.75-3)			
DAN -ve	4 (3–5)	1.33 (1-1.67)			
p	0.044*				

*ICC* Intraclass correlation coefficient, *DAN +ve* Diabetic subjects with diaetic autonomic neuropathy, *DAN -ve* Diabetic subjects without diabetic autonomic neuropathy. Data are presented as median (Interquartile range)

* Significant. ** After we had removed questions (16–19) about diarrhea from the gastrointestinal subdomain, the internal consistency of the remaining subdomain questions became acceptable (α = 0.711 and ω = 0.728). Data are presented as median (IQR)

A significant inverse correlation was observed between the total COMPASS-31 score and objective measures of parasympathetic function, specifically the 30:15 ratio (r_s_=-0.570, *p* < 0.001) and the E: I ratio (r_s_=-0.378, *p* = 0.016), indicating that higher symptom burden corresponds to reduced heart rate variability (Table 3). Among adrenergic tests, the diastolic blood pressure response to sustained handgrip showed a significant negative correlation with total COMPASS-31 score (r_s_ = -0.500, *p* = 0.001).

**Table 3:** Correlation between COMPASS-31 scores and autonomic function tests (*n* = 40)

Test	COMPASS-31						
	OI	VM	SM	GI	BL	PM	Total
Ewing battery							
30:15	-0.499 (0.001*)	-0.280 (0.080)	-0.209 (0.197)	-0.251 (0.119)	-0.362 (0.022*)	-0.139 (0.393)	-0.570 (< 0.001*)^a^
E/I	-0.448 (0.004*)	-0.153 (0.347)	-0.178 (0.273)	-0.080 (0.623)	-0.034 (0.836)	0.096 (0.555)	-0.378 (0.016*)^a^
VR	-0.280 (0.080)	0.037 (0.820)	-0.079 (0.628)	-0.012 (0.940)	-0.038 (0.814)	-0.236 (0.143)	-0.211 (0.191)
SBP-AS	0.420 (0.007*)	0.066 (0.687)	0.031 (0.849)	0.239 (0.138)	0.010 (0.951)	0.316 (0.047*)	0.403 (0.010*) ^a^
DBP-SHG	-0.447 (0.004*)	-0.343 (0.030*)	-0.381 (0.015*)	-0.153 (0.345)	-0.250 (0.119)	0.027 (0.870)	-0.500 (0.001*) ^a^
SSR							
Absence	0.222 (0.169)	0.203 (0.210)	0.332 (0.037*)	0.292 (0.067)	0.076 (0.641)	0.256 (0.111)	0.333 (0.036*)
UL av-Amp	-0.266 (0.097)	-0.101 (0.535)	-0.200 (0.215)	-0.200 (0.215)	-0.030 (0.856)	-0.140 (0.387)	-0.316 (0.048*)
LL av-Amp	-0.072 (0.657)	-0.181 (0.262)	-0.282 (0.078)	-0.282 (0.078)	-0.160 (0.325)	-0.195 (0.227)	-0.265 (0.098)

*OI* Orthostatic intolerance, *VM* Vasomotor, *SM* Secretomotor, *GI* Gastrointestinal, *BL* Bladder, *PM* Pupillomotor, *E/I* Expiratory to inspiratory ratio, *VR* Valsalva ratio, *SBP-AS* Systolic blood pressure after active standing, *DBP-SHG* Diastolic blood pressure after sustained hand grip, *SSR* Sympathetic skin response, *UL* Upper Limb, *av-Amp* Average amplitude

* Significant, ^a^ significant findings that survived the Benjamini-Hochberg false discovery rate correction. Data are presented as Spearman’s rank “r_s_” “for all data”, or point biserial “r_pb_” correlation coefficient “for SSR Absence” (p value)

The total COMPASS-31 score demonstrated significant positive correlations with somatic neuropathy severity as assessed by the UENS (r_s_=0.517, *p* < 0.001) and neuropathic pain intensity assessed by the NRS (r_s_=0.495, *p* = 0.001) (Table 4). Furthermore, a significant negative correlation was found between the COMPASS-31 score and the EQ-5D-5 L index (r_s_=-0.539, *p* < 0.001), confirming the impact of autonomic symptom burden on quality of life.

**Table 4:** Correlation between Total COMPASS-31 scores and somatosensory and quality of life scales (*n* = 40)

Test	Total COMPAS-31
UENS	0.517 (< 0.001*)
NRS	0.495 (0.001*)
EQ-5D-5 L index	-0.539 (< 0.001*)

*UENS* Utah early neuropathy scale, *NRS* 11-item numeric pain scale, *EQ-5D-5 L* EuroQoL-5 Dimensions- 5 Levels.

Data are presented as Spearman’s rank “r_s_” correlation coefficient (p value)

**Fig. 2:**
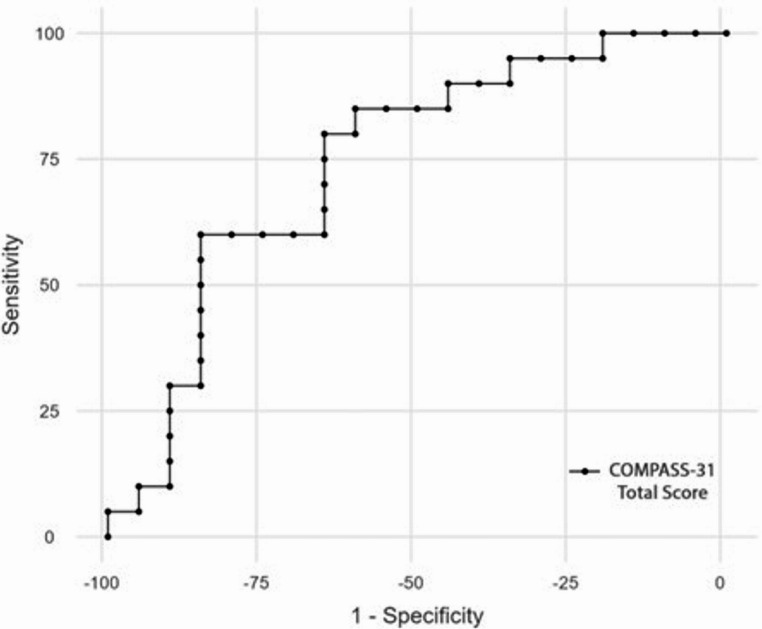
Receiver operator characteristics (ROC) curve of COMPASS-31 total score in stratifying patients with confirmed DAN**. **Diagnostic accuracy was fair (AUC = 0.742, *p* = 0.003, 95% confidence interval 0.580 to 0.867). Based on the curve coordinates, the optimal cut-off was > 28.429, with a sensitivity of 80% and specificity of 65%. A cut-off of > 36.738 yielded a sensitivity of 60% and a specificity of 85%. To increase screening performance, a cut-off of > 18.56 yielded a sensitivity of 95% and a specificity of 35%

ROC curve analysis yielded an AUC of 0.742 (95% CI: 0.580–0.867; *p* = 0.003) for identifying DAN (Fig. 2). The optimal balanced cut-off was identified at > 28.43 (Sensitivity 80%, Specificity 65%)13. A lower cut-off of > 18.56 increased sensitivity to 95% (Specificity 35%), favoring its use for screening, whereas a higher cut-off of > 36.74 improved specificity to 85% (Sensitivity 60%) for diagnostic confirmation.

## Discussion

This study developed and validated an Arabic version of the COMPASS-31 (A-COMPASS-31) in diabetic patients with DAN, representing the first comprehensive psychometric evaluation of COMPASS-31 in an Arabic-speaking cohort of patients. The results demonstrate that the A-COMPASS-31 maintains excellent psychometric properties comparable to the original English version, establishing it as a reliable and valid tool for assessing and screening autonomic symptoms in an Arabic-speaking Egyptian population, given the unique linguistic and cultural considerations intrinsic to this population.

The A-COMPASS-31 demonstrated excellent internal consistency in our cohort, consistent with the original validation by Sletten and colleagues [10] and other subsequent linguistic validations. The original COMPASS-31 development study reported strong correlation with the longer COMPASS instrument (*r* = 0.95) while maintaining brevity and clinical feasibility [10]. Our findings corroborate the psychometric robustness observed in other linguistic validations [16, 17, 20]. The consistency of these psychometric properties across diverse linguistic and cultural contexts supports the cross-cultural validity of the COMPASS-31 construct.

It is important to acknowledge that domain-level reliability can vary across translations. In our study, the secretomotor domain demonstrated low internal consistency (α = 0.526), consistent with findings from the original English validation (α = 0.246) [11], the original Mayo Clinic study (α = 0.71) [10], and the German validation (α = 0.676) [16]. This low consistency appears inherent to the secretomotor domain items in the COMPASS-31 instrument, which combines assessment of sicca symptoms (dry eyes, dry mouth) and sweating abnormalities. In the original factor analysis, the sweating question was not retained due to weak clustering with sicca items yet was retained in the final COMPASS-31 for clinical relevance [10]. This domain heterogeneity may reflect cultural differences in symptom perception, reporting patterns, or genuine differences in autonomic dysfunction profiles across populations. The removal of gastrointestinal diarrhea items (questions 16–19) that we explored improved gastrointestinal domain internal consistency (α = 0.615 to 0.711) suggesting that local dietary habits or non-diabetic gastrointestinal etiologies [34, 35] may confound specific symptom items in our Egyptian population, but this removal was not implemented in the final version to maintain structural fidelity to the original COMPASS-31, enabling international comparison of results.

A critical aspect of our validation was the correlation between COMPASS-31 scores and objective autonomic function tests, including the Ewing battery and SSR. Our results demonstrated only moderate correlations between COMPASS-31 and objective measures, consistent with other linguistic validation studies [13, 17, 36]. This discordance between subjective and objective autonomic severity has been documented extensively and recently, with a large retrospective study by Ruška and colleagues [36] and a separate study by Novak and colleagues [37]; both studies found no meaningful correlation between COMPASS-31 and composite objective autonomic severity scores across a broad referral population. This finding highlights that COMPASS-31 may capture more autonomic domains than what is routinely tested on autonomic reflex testing (i.e., not just cardiovascular and sudomotor) and can thus more meaningfully capture symptom burden and quality of life impact.

COMPASS-31 performance in diabetes has been evaluated across multiple cohorts with variable results and cutoff values suggested in defining CAN. The A-COMPASS-31 score carried good sensitivity (80%) but only modest specificity (65%) when using an optimal cutoff score > 28.43 in identifying DAN, which renders it insufficient for standalone diagnostic use. While scores above 36.74 offer higher diagnostic certainty (85% specificity), any score exceeding the optimal cut-off of 28.43 should ideally prompt confirmatory objective autonomic testing. Greco and colleagues [17], in the Italian validation in diabetes, recommended cutoffs of 16 for early CAN (sensitivity 75.0%, specificity 64.9%) and 17 for confirmed CAN (sensitivity 70%, specificity 66.7%). Singh and colleagues [13] reported an optimal cutoff of 28.7 for definite CAN in their Indian tertiary care sample (sensitivity 77.8%, specificity 71.7%), while Meling and colleagues, in a Norwegian digital implementation study, suggested lowering the threshold to 10 to increase screening sensitivity from 33% to 83%, whilst accepting a reduced specificity of 55% [12]. These varied cutoffs reflect important considerations regarding the intended use of COMPASS-31. As a screening tool, lower thresholds maximize sensitivity to avoid missing cases, while diagnostic applications may require higher specificity. Our study contributes to this discourse by providing performance characteristics specific to an Arabic-speaking diabetic population, which may inform region-specific screening algorithms.

Our findings assessing correlation between COMPASS-31 and neuropathy severity measures (UENS) and quality of life (EQ-5D-5 L) are clinically significant. These correlations demonstrate that autonomic symptom burden, as captured by COMPASS-31, has tangible impacts on overall neuropathy severity and patient quality of life. This convergent validity with established patient-reported outcomes strengthens the clinical relevance of COMPASS-31 scores. The relationship between COMPASS-31 and neuropathic pain (NRS) is particularly noteworthy. While autonomic and sensory symptoms are distinct manifestations of diabetic neuropathy [38], they often coexist and may share common pathophysiological mechanisms; perhaps it may be looked at as a continuum of symptoms that arise from subsequent affliction of nerve fibers [38]. Thus, comprehensive neuropathy assessment should address both sensory and autonomic domains, as both contribute to disease burden and quality of life impairment.

Our sample size of 40 subjects is modest and may limit the precision and generalizability of our findings. However, sensitivity power analysis indicated that the study was adequately powered for its primary convergent validity correlations, between-group comparison, and ROC analysis. However, our proposed cut-off values should be interpreted with caution until confirmed in a larger external cohort, as we derived our ROC cut-offs and the assessed accuracy within the same dataset without external validation. Splitting our cohort into separate groups for such validation purposes would have severely reduced statistical power. Thus, our reported diagnostic thresholds remain preliminary. Future studies in independent external cohorts are required to externally validate these cutoff values.

From a linguistic standpoint, the Arabic language presents unique translational challenges due to its rich vocabulary, regional dialects, and cultural contexts across which it is spoken in the Arab world (and beyond). Our translation was performed in an Egyptian cohort of Arabic-speaking subjects using Modern Standard Arabic, which is the formal written and educational register across Arabic-speaking countries, and which accordingly, provides a common linguistic baseline for the questionnaire.… However, our data are derived from a single center in Egypt, and dialectal as well as cultural variability across the Arab world may influence symptom expression and item interpretation. Therefore, external validity to broader regions of the Arabic-speaking world may be limited. Future studies should evaluate performance across different Arabic-speaking regions and across different dialects using multi-center validation studies. In addition, cultural factors could influence symptom reporting in Arabic speaking populations, such as communication styles, health literacy, and attitudes toward discussing bodily functions. These factors may very well affect COMPASS-31 scores and should be considered in future clinical interpretation and validation studies.

In conclusion, the present study successfully validates the A-COMPASS-31 as a reliable and valid instrument for screening and assessing autonomic symptoms in patients with DAN. The A-COMPASS-31 psychometric properties show acceptable internal consistency as well as significant correlations with neuropathy severity scales (UENS and NPS) and quality of life measures (EQ-5D-5 L). These findings support the use of A-COMPASS-31 as a practical screening tool for autonomic dysfunction in Arabic-speaking populations, especially diabetic subjects. Our study demonstrates the utility of the questionnaire as a screening and triage tool to identify patients who warrant further dedicated objective autonomic evaluation. The moderate correlations between COMPASS-31 and objective autonomic and neuropathy measures observed in our study and others reflect the complementary nature of subjective and objective assessments. Future research should include larger multicenter studies across diverse Arabic-speaking populations, longitudinal assessment of test-retest reliability and responsiveness to change, as well as evaluation of prognostic value for clinical outcomes.

## Supplementary Information

Below is the link to the electronic supplementary material.

Supplementary Material 1

## Data Availability

The datasets generated and analyzed during the current study are not publicly available due to institutional limitations, yet they are available from the corresponding author on reasonable request.
