# Supplementary material for: Validation of the Arabic version of the composite autonomic symptom score 31 questionnaire in diabetic autonomic neuropathy
Source: Neurol Sci. 2026 Mar 27;47(4):381. doi: 10.1007/s10072-026-08990-w (PMC13021850; doi:10.1007/s10072-026-08990-w)
Supplement: Supplementary file 1 — Supplementary Material 1 [file 10072_2026_8990_MOESM1_ESM.docx]

**Supplementary Table S1:** Sensitivity and post-hoc power analysis results for the A-COMPASS-31 validation study (n=40, α=0.05, two-tailed).

| **Analysis** | **Effect size** | **95% CI** | **Achieved power** |
| --- | --- | --- | --- |
| Total A-COMPASS-31 vs. UENS | r_s_ = 0.517 | [0.245, 0.714] | 93.6% |
| Total A-COMPASS-31 vs. NPS | r_s_ = 0.495 | [0.217, 0.699] | 91.0% |
| Total A-COMPASS-31 vs. EQ-5D-5L | r_s_ = −0.539 | [−0.728, −0.273] | 95.6% |
| ROC AUC (vs. null 0.5) | AUC = 0.742 | [0.580, 0.867] | 86.6% |
| Total A-COMPASS-31 group comparison (DAN +ve vs. DAN −ve) | Cohen's d = 0.891 | [0.241, 1.541] | 78.4% |
| **Sensitivity: minimum detectable r at 80% power** | **r = 0.431** | — | — |
| **Sensitivity: minimum detectable AUC at 80% power** | **AUC = 0.726** | — | — |
| **Sensitivity: min detectable d at 80% power** | **d = 0.909** | — | — |

UENS: Utah Early Neuropathy Scale; NPS: Neuropathic Pain Scale; EQ-5D-5L: EuroQol-5 Dimensions-5 Levels; ROC: receiver operating characteristic; AUC: area under curve; DAN: diabetic autonomic neuropathy
